# Optimal control problem arising in mathematical modeling of cerebral vascular pathology embolization

**DOI:** 10.1038/s41598-022-05231-w

**Published:** 2022-01-25

**Authors:** Tatiana Sharifullina, Alexander Cherevko, Vladimir Ostapenko

**Affiliations:** Lavrentyev Institute of Hydrodynamics of the Siberian Branch of the Russian Academy of Sciences, Novosibirsk, 630090 Russia

**Keywords:** Applied mathematics, Blood flow, Vascular diseases

## Abstract

Arteriovenous malformation (AVM) of the brain is a congenital vascular abnormality, in which the arterial and venous blood pools are intertwined and directly connected. This dangerous disease causes a high risk of intracranial hemorrhage and disrupts brain functioning. The preferred method of AVM treating is embolization, which is the endovascular filling of abnormal AVM vessels with a special embolic agent. Despite the fact that this method is widely used in neurosurgery, in some cases its use is accompanied by perioperative AVM vessels rupture. In this regard, the aim of this work is to study the optimal scenarios for multi-stage AVM embolization from the effectiveness and safety of the procedure point of view. Mathematically, the joint movement of blood and embolic agent in the AVM body is described on the basis of a one-dimensional two-phase filtration model, which takes into account the redistribution of blood to surrounding healthy vessels. For the numerical solution of the resulting integro-differential system of equations, a monotonic modification of the CABARET scheme is used. To find optimal embolization scenarios, the optimal control problem with phase constraints arising from medicine is formulated. A modified particle swarm optimization method is used to solve this problem numerically. This technique is used to obtain optimal embolization scenarios on the basis of real patients clinical data collected during neurosurgical operations.

## Introduction

Arteriovenous malformation (AVM) of the brain is a congenital vascular abnormality, in which the arterial and venous blood pools are intertwined and directly connected, bypassing the capillary vessel network. By type of pathological blood vessels, connecting the arterial and venous pools, AVMs can be divided into racemic pathology (consisting of large number of small diameter vessels, which are chaotically intertwining and intersecting with each other) and fistula one (consisting of large vessels). The resistance of the corresponding part of circulatory system is reduced due to the capillary network absence. This causes changes in both the hemodynamic parameters (flow rate and pressure) and the strength properties of the blood vessels^1–4^.

Currently, the most common methods of AVM treatment are microsurgical, endovascular and radiosurgical^5^. AVMs have a complex effect on intracranial hemodynamics, which varies with changes in morphology and angioarchitecture, especially with different treatment methods. Endovascular embolization is the filling of abnormal blood vessels with a special embolic agent in order to shut them off from the bloodstream. Due to the minimal invasiveness and the possibility of operating in deep, functionally significant brain areas, embolization is one of the most effective methods at the current level of medical development^6^. Despite the fact that this method is widely used, the risk of perioperative vascular rupture is still a serious danger^7^. Therefore, the study of optimal scenarios for multi-stage AVM embolization from the effectiveness and safety of neurosurgical surgery point of view remains an urgent task.

For the mathematical description of blood flow, a one-dimensional approximation of the Navier–Stokes equations is widely used, obtained by averaging these equations over blood vessel cross-section^8^. There are works in which the equations describing flows in pipes with rigid and elastic boundaries are analytically investigated^8–10^. Viscous fluid flow in a network of soft tubes is also modeled on the basis of a one-dimensional approximation of mass and momentum conservation laws^11–13^. Numerical simulation of hemodynamics for large blood vessels based on 3D–1D coupled flow is also considered^14–16^.

The interaction of AVM and blood flow in the surrounding vessels is often studied in analogy with electric and hydraulic networks^17–19^. Such models allow us to assess the impact of various embolization scenarios on blood flow redistribution and are consistent with the general medical view on pathology hemodynamics. Various approaches are used to describe the joint flow of blood and embolic agent, for example, a two-phase flow model was used to simulate a viscous fluid drop movement of embolic agent through a bifurcation point or multifurcation point based on dimensionless Navier–Stokes equations for incompressible liquids^20^. A model of AVM embolization was proposed based on the concept of two-fluid modeling and scalar transport, where embolic agent and blood interaction along with its hardening is simulated by viscosity increase^21^. Another approach to the description of the embolization process is based on the two-phase filtration model in the authors’ work^22^. The present paper is a extension of this work. In comparison to the present work in^22^ blood flow into the adjacent healthy vessels was not considered, the cross-section area and porosity were assumed to be constant, and embolization was modeled without taking into account embolic agent solidification between the stages of surgery (simplified one-stage case). The optimal control problem^22^ differs in form from the one described below, but is essentially the same, namely, it requires performing the operation in the most efficient way in compliance with safety constraints. The formulation of the optimal control problem in present paper is obtained from the control problem^22^ by interchanging the constraint and the objective functional, which is typical of extremum search problems with constraints. In the work^22^, the search for an extremum of the functional was based on the calculation of its value on the grid in the three-dimensional control parameter space with subsequent interpolation. While present work uses the global optimization method - the particle swarm method, which significantly accelerates calculations, increases their accuracy and allows us to explore the control parameter space of a larger dimension. Despite the large number of publications dedicated to the AVM hemodynamics, research expansion in this direction is a great scientific interest.

In this paper, mathematical modeling of AVM embolization is carried out as follows: the flow of blood and embolic agent through the AVM body is considered as filtration flow based on Darcy’s law, taking into account the redistribution of blood to healthy vessels. This approach is justified for small-vascular racemic AVM compartments embolization description. It is assumed that both liquids are Newtonian with constant viscosity, incompressible and immiscible, while ignoring the interfacial capillary forces, the effect of embolic agent adsorption, as well as the deformation and permeability of the vessel walls.

As a result, the mathematical description of the joint movement of blood and embolic agent in the AVM body is based on a one-dimensional two-phase filtration model that takes into account the redistribution of blood to surrounding healthy vessels. For the numerical solution of the resulting system of integro-differential equations, a monotonic modification of the CABARET scheme is used, which localizes with high accuracy the strong and weak discontinuities that occur when solving this problem^23,24^. For numerical calculations real blood and embolic agent (ONYX18) viscosities were used. To find optimal embolization scenarios, the optimal control problem of multi-stage embolization with phase constraints arising from medicine is formulated. To solve this problem numerically a particle swarm optimization method is used^25,26^, which is specially modified in order to adapt it to the problem under consideration. The proposed technique is used to obtain optimal embolization scenarios on the basis of real patients clinical data collected during neurosurgical operations^22,27^.

In the next section, a mathematical formulation of the one-stage embolization problem is given, leading to an initial-boundary value problem for a system of integro-differential equations, including a hyperbolic partial differential equation, which is a quasilinear scalar conservation law with a nonconvex flux. Then the optimal control problem of one-stage AVM embolization is formulated, where the control is a time function setting the boundary condition of the problem and is included in the coefficients of the equation. The objective functional is the integral of the initial-boundary value problem solution at terminal time. The phase constraints on the control function represent additional integral and algebraic relations. The objective functional and constraints are selected according to medical indications. We introduce a class of piecewise linear continuous and discontinuous functions used as controls for the AVM embolization process. Further, the optimal control problem of multi-stage AVM embolization is formulated, which leads to a chain of integro-differential initial-boundary value problems, in which each subsequent problem uses the results of solving the previous problem as parameters of a new system of equations.

Next, we present a monotonic modification of the CABARET scheme, which localizes with high accuracy the strong and weak discontinuities that arise when solving the initial-boundary value problem under consideration. The modification of the particle swarm optimization method is proposed that is specially adapted to the considering problem in order to more fully investigate the admissible parameters area and accelerate the progress of the particle swarm to the absolute minimum. Numerical verification of the optimal control problem of multi-stage embolization using real patients clinical data was performed, on the basis of which AVM embolization optimal scenarios were obtained. Also we discusses the limitations of the proposed method, as well as the possibilities for its further development.

## One-stage embolization problem formulation

**Figure 1:**
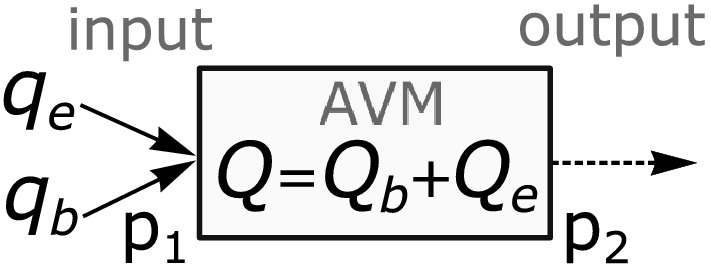
AVM embolization model representation. *Q*—volume flow rate of two-phase mixture; $Q_b$ and $Q_e$—volume flow rates of blood and embolic agent inside AVM; $q_b$ and $q_e$—volume flow rates of blood and embolic agent at the AVM input; $p_1$—blood pressure at the AVM input; $p_2$—blood pressure at the AVM output.

In this paper, blood and embolic agent movement through small-vascular racemic AVM is modeled (Fig. 1) as a two-phase filtration process of immiscible incompressible liquids, where the displaced phase is blood, and the displacing phase is embolic agent. We also take into account the redistribution of blood to surrounding healthy vessels. We will consider a one-dimensional case, which mathematical description is based on Darcy’s law and the mass conservation laws for each phase. In the one-stage embolization case, an initial-boundary value problem is obtained for a system of integro-differential equations, in which the hyperbolic partial differential equation is a quasi-linear scalar conservation law with a nonconvex flux function. This conservation law is similar to the one widely used to describe the multiphase filtration process^28,29^.

Based on this initial-boundary value problem, it is set the task of finding the optimal regime of one-stage AVM embolization from the effectiveness and safety of neurosurgical surgery point of view. The objective functional and constraints that arise in the optimal control problem are selected according to medical indications. The control is a time function that determines the embolic agent injection at the AVM input. The embolization effectiveness is determined by the degree of blood displacement from the AVM by the embolic agent at the operation end. One of the possible risks of the operation complications is the embolic agent entering in the vein, as a which result the AVM or vein vessels rupture may occur. Also, during the operation, vascular network has an additional stress, since when the embolic agent is injected, the pressure in the arterial vessels increases. Excessive pressure build-up is dangerous. Both of these factors increase the brain hemorrhage risk^30^ and will be further used in the optimal embolization control problem.

### Derivation of the conservation law simulating the process of one-stage embolization

Consider the one-dimensional joined filtration flow of blood and embolic agent through the AVM body. Denote by *A* the AVM cross-section area available for the phase flows, by *m* the AVM body porosity, by *Q* the volume flow rate of two-phase mixture. These values can vary both in space and in time. Let $S(t,x)\in [0,1]$ be the local saturation (blood concentration) of the porous medium by the displaced phase (i.e. the fraction of the pore volume occupied by blood). Taking this into account, the concentration of the displacing phase is $1-S(t, x)$. We introduce the Buckley–Leverett saturation function *f*(*S*)^29^, which sets the distribution of the phases volume flow inside a porous medium and is equal to the flow fraction of displaced phase in the total two phases flow.

We derive the differential form of the integral conservation law of blood volume, assuming that the AVM walls are impermeable and the phases are incompressible fluids. The change in blood volume $\Delta V_1$ on the segment $\Delta x=x_2-x_1$ during the time $\Delta t=t_2-t_1$ is

1 $$\begin{aligned} \Delta V_1=\int _{x_1}^{x_2}{m\,A\,S}|_{t_1}^{t_2}\, dx. \end{aligned}$$

This change is due to the blood flow through the cross-sections at points $x=x_1$ and $x=x_2$:

2 $$\begin{aligned} \Delta V_2=-\int _{t_1}^{t_2}{Q\,f(S)}|_{x_1}^{x_2}\, dx. \end{aligned}$$

Equating the values (1) and (2), we obtain the integral conservation law of blood volume

3 $$\begin{aligned} \int _{x_1}^{x_2}{m\,A\,S}|_{t_1}^{t_2}\, dx+\int _{t_1}^{t_2}{Q\,f(S)}|_{x_1}^{x_2}\, dx=0, \end{aligned}$$

where *m*(*x*) and *A*(*x*) are given piecewise continuous functions of the spatial coordinate *x*. The differential form of the conservation law (3) has the form

4 $$\begin{aligned} \frac{\partial }{\partial t}\left( m\,A\,S\right) +\frac{\partial }{\partial x} \left( Q\,f(S)\right) =0. \end{aligned}$$

Since the Eq. (4) is hyperbolic, its solution *S*(*t*, *x*) is considered in the class of piecewise continuous functions. We will assume that the Buckley–Leverett function $f(S)\in C^2[0,1]$ satisfies the following conditions

$$\begin{aligned} f(0)= & {}\ 0, \ f(1)=1; \quad f'(S)>0, \ S\in (0,1);\\ f''(S)> & {}\ 0, \ S\in (0,\xi ); \quad f''(S)< 0, \ S\in (\xi ,1); \end{aligned}$$

where $\xi$ is the inflection point. Since the AVM side surface is impermeable and the liquids are incompressible, the total volume flow rate of the two phases *Q*(*t*) does not depend on the spatial coordinate *x* and is determined by the relation

$$\begin{aligned} Q(t)=Q_b(t,x)+Q_e(t,x), \end{aligned}$$

where $Q_b=Q\,f(S)$ is the volume blood flow and $Q_e=Q\,(1-f(S))$ is the volume flow rate of an embolic agent inside AVM. With this assumptions, the Eq. (4) can be written in the following equivalent non-divergent form

5 $$\begin{aligned} \frac{\partial S}{\partial t}+\frac{Q}{m\,A} \, \frac{\partial f(S)}{\partial x}=0, \end{aligned}$$

which is more convenient for performing numerical calculations.

### Initial and boundary conditions

The differential equation (5) is solved in the domain

$$\begin{aligned} W=\left\{ (x,t):\ \ 0\le x \le L,\ \ 0\le t \le T \right\} , \end{aligned}$$

where *L* and *T* are some positive numbers. Since at the initial time $t=0$ there is only blood inside the AVM, then the initial value of its concentration is given by the following formulas

6 $$\begin{aligned} S(0,x)=1, \ x\in [0,L]. \end{aligned}$$

Blood flow changes during the cardiac cycle is not taken into account due to the duration of the embolization process is significantly longer than the cardiac cycle duration, and the blood flow rate $q_b(t)=Q_b(t,0)$ at the AVM input is determined by the formula

$$\begin{aligned} q_b(t)={\bar{q}}_b({\bar{S}}(t)), \end{aligned}$$

in which ${\bar{q}}_b({\bar{S}})$ is a given function that determines the redistribution of blood between AVM and surrounding healthy vessels, where

7 $$\begin{aligned} {\bar{S}}(t)=\dfrac{1}{L} \int \limits _0^L{S(t,x) dx}, \end{aligned}$$

is the average blood concentration in the AVM. At the AVM input ($x=0$) the embolic agent flow $q_e(t)=Q_e(t,0)$ determines the embolization process and is a control function. Then the flow rate *Q*(*t*) of two-phase mixture inside the AVM, included in (5), is determined by the formula

8 $$\begin{aligned} Q(t)=q_e(t)+q_b(t)= q_e(t)+{\bar{q}}_b\left( {\bar{S}}(t)\right) . \end{aligned}$$

Assuming that the blood and embolic agent flows satisfy the inequalities

$$\begin{aligned} q_b(t)>0, \quad q_e(t)\ge 0, \quad t\in [0,T], \end{aligned}$$

we obtain that the flow of two-phase mixture

$$\begin{aligned} Q(t)>0, \quad t\in [0,T], \end{aligned}$$

in consequence, the characteristics of the differential equation (5) propagate with velocities $Q(t)f'(S)/\left( m(x)\,A(x) \right) >0$. This means that for the correct formulation of the initial-boundary value problem for the Eq. (5) one boundary condition must be set on the left boundary of the segment [0, *L*] and there is no need to set boundary conditions on the right boundary of this segment. It follows from formula $f(S(t,x))=Q_b(t,x)/Q(t)$ and relation (8) that the boundary value *S*(*t*, 0) is expressed in terms of boundary phase flows and is defined as follows

9 $$\begin{aligned} f(S(t,0))=\frac{{\bar{q}}_b({\bar{S}}(t))}{q_e(t)+{\bar{q}}_b({\bar{S}}(t))}, \end{aligned}$$

where the function ${\bar{S}}(t)$ is given by the integral formula (7). As a result, we obtain the integro-differential problem (5), (6), (8), (9) in which the integral relation (9) plays the role of a non-standard boundary condition for the function *S*(*t*, *x*). It is important to emphasize that the control function $q_e(t)$ is included both in the coefficients of the Eq. (5) and in the boundary condition (9).

## Optimal control problem in the case of one-stage embolization

To formulate the embolization optimal control problem, it is necessary to determine objective functional and constraints arising from medical indications, which establish the effectiveness criteria and safety conditions of the operation. The embolization effectiveness will be determined by how completely the embolic agent fills the AVM at the operation end. Mathematically, this condition lead to minimizing the objective functional

10 $$\begin{aligned} J[q_e]=\dfrac{1}{L}\int \limits _0^LS_{q_e}(T,x)dx, \end{aligned}$$

where $S_{q_e}(t,x)$ is the solution of the initial-boundary value problem (5), (6), (8), (9) with control function $q_e(t)$, which sets the embolic agent flow at the AVM input.

The safety embolization conditions can be chosen in the form of the following restrictions

11 $$\begin{aligned} S_{q_e}(t,L)= & {} \ 1, \quad t\in [0,T], \end{aligned}$$

12 $$\begin{aligned} \max _{t\in [0,T]} p_1(t)\le & {}\ \ p_{*}, \end{aligned}$$

where $p_{1}$ is the pressure at the AVM input, and $p_{*}$ is the specified critical pressure. Fulfillment the condition (11) avoids the penetration of the embolic agent into the venous bed, and fulfillment the inequality (12) avoids an excessive increase in blood pressure in the AVM. Violation of the conditions (11) and (12) increases the cerebral hemorrhage risk^30^.

Taking into account the fact that $Q_b=Q\,f(S)$, the pressure function is determined from Darcy’s law for AVM^29^

13 $$\begin{aligned} p_1(t)=p_2({\bar{S}}(t))+Q(t)\int \limits _0^Lr_b(x,S(t,x))f(S(t,x))dx, \end{aligned}$$

where $p_2({\bar{S}})$ is a given function of the blood pressure at the AVM output. The local resistance to blood flow inside the AVM is given by the ratio

$$\begin{aligned} r_b(S)=\dfrac{\eta _b}{A(x)\,K\,k_b(S)}, \end{aligned}$$

where $\eta _b$ is the blood viscosity, *K* is the absolute permeability of the AVM and $k_b(S)$ is the blood relative phase permeability; the values of $\eta _b$, *K* and $k_b(S)$ are assumed to be known. For the given functions *f*(*S*), $r_b(S)$, ${\bar{q}}_b({\bar{S}})$ and $p_2({\bar{S}})$, Eqs. (5), (8) and (13) form a closed system of integro-differential equations for determining the blood concentration *S*(*t*, *x*) inside the AVM, the mixture flow rate *Q*(*t*) and the pressure $p_1(t)$ at the AVM input. The pressure $p_1(t)$ is used to verify the considered optimal AVM embolization model by comparing with the pressure obtained during intraoperative monitoring.

Thus, in the optimal control problem, it is necessary to find a function $q_e(t)$ that minimizes the objective functional (10), which is determined on the basis of the problem (5), (6), (8), (9) solution. Besides, it must be done the restrictions (11), (12) on the blood concentration *S* and the pressure $p_1(t)$ determined by the formula (13).

## Control functions class

We consider a numerical solution of the problem (5), (8), (13) with initial (6) and boundary (9) conditions that satisfies the constraints (11) and (12). The choice of the control class is determined by one of the methods of embolization in real surgical practice: at the initial time interval, there is increase in the embolic agent supply; at the next time interval, a constant value of this flow is maintained; and at the last short time interval, there is a decrease in the embolic agent supply to the AVM input. We use linear interpolation of this embolization method. As a result, we consider the following continuous functions as a control function class

14 $$\begin{aligned} q_e(t)=\gamma \, Q(0)\,E(t), \end{aligned}$$

where $\gamma >0$ is a dimensionless parameter, $Q(0)=q_b(0)$ is the blood flow through the AVM before surgery,

15 $$\begin{aligned} E(t)=\left\{ \begin{array}{ll} t/\theta , &{}\quad 0\le t\le \theta , \\ 1, &{}\quad \theta \le t\le T-\varepsilon ,\\ (T-t)/\varepsilon , &{}\quad T-\varepsilon \le t\le T , \end{array} \right. \end{aligned}$$

is a dimensionless function in which the parameter $\theta$ satisfies the condition $0<\theta \le T-\varepsilon$, and $\varepsilon \in (0,T)$ is a small constant. Admissible control class is expanded to include the function that is discontinuous at the initial moment of time

16 $$\begin{aligned} E(t)=\left\{ \begin{array}{ll} 0, &{}\quad t=0, \\ 1, &{} \quad 0<t\le T-\varepsilon ,\\ (T-t)/\varepsilon , &{}\quad T-\varepsilon <t\le T , \end{array} \right. \end{aligned}$$

obtained from the function (15) when $\theta \rightarrow 0$.

For the control class (14) and (15), time interval $[0,\theta ]$ corresponds to an increase in the supply of embolic agent to the value $\gamma \, Q(0)$; time interval $[\theta ,T-\varepsilon ]$ corresponds to maintaining a constant value of this flow; time interval $[T-\varepsilon ,T]$ corresponds to a decrease in the embolic agent supply to the AVM input. The control class (14) and (16) corresponds to the case when there is a discontinuous increase in the embolic agent supply.

As a result, the optimal embolization problem is led to finding the control parameters

17 $$\begin{aligned} \gamma >0, \quad \theta \ge 0, \quad T\ge \theta +\varepsilon , \end{aligned}$$

that determine embolic agent supply (14). The corresponding solution of the initial-boundary value problem (5), (6), (8), (9), (13) gives an absolute minimum to the functional (10), which corresponds to minimizing the average blood concentration in the AVM at the operation end. The solutions to the optimal control problem must satisfy the constraints (11) and (12), where (11) prohibits the embolic agent from entering the vein, and (12) restricts the blood pressure at the entrance to the AVM during embolization. Next, instead of the parameters (17), we will use the following control parameters

$$\begin{aligned} \gamma >0, \quad \theta _1=\theta \ge 0, \quad \theta _2=T-\theta -\varepsilon \ge 0, \end{aligned}$$

where the parameter $\theta _1$ corresponds to the time of increasing the embolic agent supply to the AVM input, $\theta _2$ corresponds to the time of maintaining the achieved flow of embolic agent, $\gamma$ is the intensity of the embolic agent supply.

## Multi-stage embolization

### Multi-stage embolization problem formulation

A real neurosurgical operation for AVM embolization usually proceeds in several stages, the number of which is denoted by *N*. Mathematical modeling of multi-stage embolization is a generalization of the one-stage embolization described in the previous section. Between the stages there are time breaks, during which the embolic agent remains fixes inside the AVM and does not change its location in the following stages of embolization. For the mathematical modeling, we assume that the duration of these time breaks is zero. Taking this into account, embolization at each *i*-th stage occurs on the time interval $[T_{i-1},T_i]$, where $T_0=0$. The cross-section area available for the flow of phases at the *i*-th stage is denoted by $A_i(x)$, so $A_1=A$ is the original cross-section area. The fraction of cross-section $A_i$ occupied by blood is denoted by $S_i(t,x)$, and the fraction of the original cross-section $A_1$ occupied by blood is denoted by $\Psi _i=S_iA_i/A_1$. Between the stages there is a change in the fraction of the AVM cross section available for the flow of phases

$$\begin{aligned} A_i(x)=A_1(x)\Psi _{i-1}(T_{i-1},x)=A_{i-1}(x)S_{i-1}(T_{i-1},x), \end{aligned}$$

which establishes a connection between the successive embolization stages. We will assume that in the AVM part available for the phases flow it is not change from stage to stage the filtration characteristics: porosity *m*, absolute permeability *K*, relative phase permeability $k_b$ and Buckley–Leverett function *f*. In the multi-stage case, the embolization process at each *i*-th stage is modeled similarly to the one-stage embolization case, which was described above. Thus, the initial-boundary value problem simulating the *i*-th embolization stage on the time interval $[T_{i-1},T_i]$, is similar to (5), (6), (8), (9), (13), is given by equations

18 $$\begin{aligned}&\frac{\partial S_i}{\partial t}+\frac{Q_i}{mA_i} \, \frac{\partial f(S_i)}{\partial x}=0, \end{aligned}$$

19 $$\begin{aligned}&Q_i(t)=q_e(t)+{\bar{q}}_b\left( {\overline{\Psi }}_i(t)\right) , \end{aligned}$$

20 $$\begin{aligned}&p_1(t)=p_2({\overline{\Psi }}_i(t))+Q_i(t)\int \limits _0^Lr_b(x,\Psi _i(t,x))f(\Psi _i(t,x))dx, \end{aligned}$$

21 $$\begin{aligned}&{\overline{\Psi }}_i(t)=\dfrac{1}{L} \int \limits _0^L\Psi _i(t,x) dx, \quad r_b(x,\Psi _i(t,x))=\dfrac{\eta _b}{A_i(x)\,K\,k_b(\Psi _i(t,x))}, \end{aligned}$$

with the following initial and boundary conditions

22 $$\begin{aligned} S_i(0,x)= & {} \ 1, \quad x\in [0,L], \end{aligned}$$

23 $$\begin{aligned} f(S_i(t,0))= & {} \frac{{\bar{q}}_b({\overline{\Psi }}_i(t))}{q_e(t)+{\bar{q}}_b({\overline{\Psi }}_i(t))}. \end{aligned}$$

### Optimal control problem in the case of multi-stage embolization

For the optimal control problem of multi-stage embolization, we will use the average blood concentration in the AVM at the end of the *N*-th stage as the objective functional

24 $$\begin{aligned} J_N[q_{e}]=\dfrac{1}{L}\int \limits _0^L\Psi _N(T_N,x)\,dx, \quad \Psi _N=\dfrac{S_NA_N}{A_1}, \end{aligned}$$

where $S_N$ is the solution of problem (18)–(23) at the *N*-th embolization stage, and control function $q_e(t)$ is given on the interval $t\in [0,T_N]$. In this case, constraints similar to (11), (12) have the form

25 $$\begin{aligned} S_{i}(t,L)= & {}\ 1, \quad t\in [T_{i-1},T_i],\quad i\in \overline{1,N}. \end{aligned}$$

26 $$\begin{aligned} \max _{t\in [T_{i-1},T_i]} p_1(t)\le & {}\ \ p_{*} ,\quad i\in \overline{1,N}. \end{aligned}$$

For multi-stage embolization, we consider a control that at each stage similar to the control for one-stage embolization (14)–(16). Such control can be considered as a linear approximation of the clinical process of multi-stage embolization and written as follows

27 $$\begin{aligned} q_e(t)= & {} \gamma _i\, Q_i(T_{i-1})\,E_i(t),\ \gamma _i>0, \quad t\in [T_{i-1},T_i], \quad i=\overline{1,N}, \end{aligned}$$

28 $$\begin{aligned} E_i(t)= & {} \left\{ \begin{array}{ll} (t-T_{i-1})/\theta _i, &{}\quad T_{i-1}\le t\le T_{i-1}+\theta _i, \\ 1, &{}\quad T_{i-1}+\theta _i\le t\le T_i-\varepsilon ,\\ (T_i-t)/\varepsilon , &{}\quad T_i-\varepsilon \le t\le T_i , \end{array} \right. \end{aligned}$$

where the parameters $\theta _i$, $T_i$ and $\varepsilon$ satisfy the conditions

$$\begin{aligned} 0<\varepsilon<\min _{i=\overline{1,N}}\left( T_i-T_{i-1}\right) , \quad 0<\theta _i\le T_i-T_{i-1}-\varepsilon . \end{aligned}$$

Admissible control class is expanded to include discontinuous functions at $t=T_{i-1}$

29 $$\begin{aligned} E_i(t)=\left\{ \begin{array}{ll} 0, &{}\quad t=T_{i-1}, \\ 1, &{}\quad T_{i-1}t\le T_i-\varepsilon ,\\ (T_i-t)/\varepsilon , &{} \quad T_i-\varepsilon \le t\le T_i , \end{array} \right. \end{aligned}$$

obtained from functions (28) when $\theta _i\rightarrow 0$.

As a result, the optimal embolization problem is reduced to finding the control parameters

30 $$\begin{aligned} \gamma _i>0, \quad \theta _i\ge 0, \quad T_i\ge T_{i-1}+\theta _i+\varepsilon , \quad i\in \overline{1,N}, \end{aligned}$$

that determine the embolic agent supply (27), and the corresponding solution of the initial-boundary value problem of multi-stage embolization (18)-(23) gives an absolute minimum to the functional (24), which corresponds to minimizing the average blood concentration in the AVM at the multi-stage operation end. The solutions to the multi-stage optimal control problem must satisfy the constraints (25) and (26), where (25) prohibits the embolic agent from entering the vein, and (26) restricts the blood pressure at the entrance to the AVM at *i*-th embolization stage. Next, instead of the parameters (30) we will use the following control parameters

31 $$\begin{aligned} \gamma _i>0, \quad \theta _{1i}=\theta _i\ge 0, \quad \theta _{2i}=T_i-T_{i-1}-\theta _i-\varepsilon \ge 0, \quad i\in \overline{1,N}, \end{aligned}$$

where the parameter $\theta _{1i}$ corresponds to the time of increasing the embolic agent supply to the AVM input, $\theta _{2i}$ corresponds to the time of maintaining the achieved flow of embolic agent, $\gamma _i$ is the intensity of the embolic agent supply at the *i*-th embolization stage.

## Methods

### Finite difference scheme

For the numerical solution of the initial boundary value problem (22), (23) for the system of integro-differential equations (18)–(21), we will use a monotone modification of the explicit two-layer time the CABARET scheme^31^, which has a second-order approximation on smooth solutions. This modification of the CABARET scheme was proposed in paper^23^ and is used to model the problems of hemodynamics^22,32^. The main advantages of this scheme are due to the fact that it is given on a compact spatial stencil and, when approximating linear equations, is time-reversible and accurate with two different Courant numbers $r=0.5, 1$, which gives it unique dissipative and dispersive properties^33^.

Numerical simulation is carried out one after another at all stages of embolization, and the same difference scheme is applied at each stages. We present the algorithm of CABARET scheme for the *i*-th stage. Let us divide the segment [0, *L*] on which the considered problem is solved into *J* equal parts of length $h=L/J$ and define a rectangular uniform difference grid

32 $$\begin{aligned} \{x_j,t_i^n\}: \ x_j=jh, \ j=\overline{0,J}; \quad t^{n+1}_i=t^n_i+\tau ^n_i, \ t^0_i=0; \end{aligned}$$

where *h* is a constant spatial step, and $\tau ^n_i$ is the time step at the *n*-th time layer, determined from the CFL-stability condition at the *i*-th embolization stage. Let us denote by $S_i^c$ the approximate numerical value of the blood concentration $S_i$ at the *i*-th stage. The CABARET scheme uses the flux $(u_i)_j^n=S_i^c(t_i^n,x_j)$ and conservative $(U_i)_{j+1/2}^n=S_i^c(t_i^n,x_{j+1/2})$ variables set respectively in the integer $x_j$ and half-integer $x_{j+1/2}=x_j+h/2$ spatial nodes of the difference grid (32).

Let $(u_i)_j^n$ and $(U_i)_{j+1/2}^n$ be the known numerical solution of the problem (18)–(23) at the time layer $t_i^n$; for $n=0$ the exact grid approximation of the initial condition (22):

$$\begin{aligned} (u_i)_j^0=1, \ \ j=\overline{0,J}; \quad (U_i)_{j+1/2}^0=1, \ \ j=\overline{0,J-1}. \end{aligned}$$

The numerical solution $(u_i)_j^{n+1}$, $(U_i)_{j+1/2}^{n+1}$ at the time layer $t_i^{n+1}$ is obtained by CABARET scheme in several stages. At the first stage, according to the formula

33 $$\begin{aligned} \tau _i^n=\dfrac{rh}{\max \limits _j a_i\left( t_i^n,x_{j+1/2}\right) }, \quad a_i(t,x)= \ \dfrac{Q_i(t)\,f'(S_i(t,x))}{m(x) \,A_i(x)}, \end{aligned}$$

where $r\in (0,1)$ is the CFL number, the time step of the scheme at the *n*-th time layer is calculated. At the second stage, by approximating the Eqs. (19)–(21) with the second order, we find at the *n*-th time layer the numerical values of the mixture flow

34 $$\begin{aligned} Q_i^n= q_e(t_i^n)+{\bar{q}}_b\left( {\overline{V}}_i^n\right) \end{aligned}$$

and the blood pressure at the AVM input

$$\begin{aligned} p(t_i^n)=p_2\left( {\overline{V}}_i^n\right) + \frac{Q_i^n\eta _b}{K} \, \sum \limits _{j=0}^{J-1} \, \frac{f\left( (V_i)_{j+1/2}^n\right) h}{A_1(x_{j+1/2}) \, k_b\left( (V_i)_{j+1/2}^{n}\right) }, \end{aligned}$$

where

$$\begin{aligned} {\overline{V}}_i^n=\frac{h}{L}\sum \limits _{j=0}^{J-1}(V_i)_{j+1/2}^n, \quad (V_i)_{j+1/2}^n=\dfrac{(U_i)_{j+1/2}^n \, A_i(x_{j+1/2})}{A_1(x_{j+1/2})}. \end{aligned}$$

At the third stage for $j=\overline{0,J-1}$ using the difference equations

$$\begin{aligned} \frac{(U_i)_{j+1/2}^{n+1/2}-(U_i)_{j+1/2}^n}{\tau _i^n/2}+ \frac{Q_i^n}{ m(x_{j+1/2}) A_i(x_{j+1/2}) } \left( \frac{(f_i)_{j+1}^n-(f_i)_j^n}{h}\right) =0, \end{aligned}$$

where $(f_i)_j^n=f\left( (u_i)_j^n\right)$, the values of the conservative variables

$$\begin{aligned} (U_i)_{j+1/2}^{n+1/2}=S_i^c \left( t_i^{n+1/2},x_{j+1/2}\right) \end{aligned}$$

at the half-integer time layer $t_i^{n+1/2}=t_i^n+\tau _i^n/2$ are calculated. At the fourth stage, the values of numerical flows

$$\begin{aligned} (f_i)_j^{n+1/2}=f\left( (u_i)_j^{n+1/2}\right) , \quad (u_i)_j^{n+1/2}=S_i^c\left( t_i^{n+1/2}, x_j\right) \end{aligned}$$

at the half-integer time layer $t_i^{n+1/2}$ are found.

At the fifth stage, using formula (34), in which the index *n* is replaced by the index $n+1/2$, the numerical value of the mixture flow $Q_i^{n+1/2}$ at the half-integer time layer is determined, after which, at the sixth stage for $j=\overline{0,J-1}$ from the difference equations

$$\begin{aligned} \frac{(U_i)_{j+1/2}^{n+1}-(U_i)_{j+1/2}^n}{\tau _i^n}+ \frac{Q_i^{n+1/2}}{m(x_{j+1/2}) A_i(x_{j+1/2})} \left( \frac{(f_i)_{j+1}^{n+1/2}-(f_i)_j^{n+1/2}}{h}\right) =0 \end{aligned}$$

we calculated the values of the conservative variables $(U_i)_{j+1/2}^{n+1}$ at the $(n+1)$-th time layer. At the seventh stage, we find the values of the flux variables $(u_i)_j^{n+1}$ at the $(n+1)$-th time layer. A detailed description of the numerical algorithm at the fourth and seventh stages is given below.

### The numerical fluxes calculations

At the beginning of the fourth stage, as a result of the second-order approximation of the integral boundary condition (23) on the left boundary of the half-integer time layer, the value of the numerical flow is calculated

35 $$\begin{aligned} (f_i)_0^{n+1/2}=\frac{{\bar{q}}_b\left( {\overline{V}}_i^{n+1/2}\right) }{ q_e(t_i^n)+{\bar{q}}_b\left( {\overline{V}}_i^{n+1/2}\right) }, \end{aligned}$$

where

36 $$\begin{aligned} {\overline{V}}_i^{n+1/2}=\frac{h}{L}\sum \limits _{j=0}^{J-1}(V_i)_{j+1/2}^{n+1/2}, \quad (V_i)_{j+1/2}^{n+1/2}=\dfrac{(U_i)_{j+1/2}^{n+1/2} \, A_i(x_{j+1/2})}{A_1(x_{j+1/2})}. \end{aligned}$$

Further in the formulas of this section, the index *i*, which specifies the number of the embolization stage, is omitted for brevity.

The numerical fluxes $f_j^{n+1/2}$ for $j=\overline{1,J}$ are defined as follows. First, their preliminary values are calculated by the formulas

$$\begin{aligned} {\bar{f}}_j^{n+1/2}=f\left( {\bar{u}}_j^{n+1/2}\right) , \ \ {\bar{u}}_j^{n+1/2}=\left( u_j^n+{\bar{u}}_j^{n+1}\right) /2, \ \ {\bar{u}}_j^{n+1}=2U_{j-1/2}^{n+1/2}-u_{j-1}^n, \end{aligned}$$

and corrected as follows

37 $$\begin{aligned} {\widetilde{f}}_j^{n+1/2}= F\left( {\overline{f}}_j^{n+1/2},m_j^n,M_j^n\right) , \end{aligned}$$

where $$m_j^n= \min \left( f_{j-1/2}^n,f_j^n\right)$$, $$M_j^n=\max \left( f_{j-1/2}^n,f_j^n\right)$$ and

$$\begin{aligned} F\left( u,m,M\right) =\left\{ \begin{array}{ll} u, &{}\quad m\le u\le M, \\ m, &{}\quad u\le m, \\ M, &{}\quad u\ge M, \end{array} \right. \end{aligned}$$

is a conventional two-sided limiter.

It is shown^34^ that the correction of fluxes (37) is not sufficient to preserve the local monotonicity of the difference solution at the $(n+1)$- th time layer, which is necessary for the absence of non-physical oscillations on the shocks. Therefore, if at the *n*-th time layer in the vicinity of the grid node $x_{k+1/2}$ the difference solution is locally monotone, an additional correction of the numerical fluxes is performed

38 $$\begin{aligned} f_{k-3/2}^n\le & {} \ f_{k-1}^n\le f_{k-1/2}^n\le f_k^n \quad \Rightarrow \quad f_k^{n+1/2}=F_1\left( {\widetilde{f}}_k^{n+1/2}, \varphi _{k-1}^n\right) , \end{aligned}$$

39 $$\begin{aligned} f_{k-3/2}^n\ge & {} \ f_{k-1}^n\ge f_{k-1/2}^n\ge f_k^n \quad \Rightarrow \quad f_k^{n+1/2}=F_2\left( {\widetilde{f}}_k^{n+1/2}, \varphi _{k-1}^n\right) , \end{aligned}$$

where

$$\begin{aligned} F_1\left( u,M\right)= & {} \left\{ \begin{array}{ll} u, &{}\quad u\le M, \\ M, &{}\quad u\ge M, \end{array} \right. \quad F_2\left( u,m\right) =\left\{ \begin{array}{ll} u, &{}\quad u\ge m, \\ m, &{}\quad u\le m, \end{array} \right. \\ \varphi _{k-1}^n= & {} \frac{A(x_{k-1}) \, m(x_{k-1}) \left( U_{k-1/2}^n-u_{k-1}^n\right) }{r^n \, Q^n}, \quad r^n=\dfrac{\tau ^n}{h}. \end{aligned}$$

If neither of the two conditions (38) or (39) is fulfilled, the flux correction ${\widetilde{f}}_k^{n+1/2}$ is not carried out, i.e. $f_k^{n+1/2}={\widetilde{f}}_k^{n+1/2}$.

At the beginning of the seventh stage, the flux variable value is found on the left boundary of the computational domain

$$\begin{aligned} u_0^{n+1}= f^{-1}\left( f_0^{n+1}\right) , \end{aligned}$$

where $f^{-1}$ is the inverse function of *f*, and $f_0^{n+1}$ is the numerical flux given by the formulas (35) and (36), in which the index $n+1/2$ is replaced by $n+1$. The flux variables $u_j^{n+1}$, where $j=\overline{1, J}$ are defined as follows: their second preliminary values are calculated by the formulas

$$\begin{aligned} {\widetilde{u}}_j^{\,n+1}=2u_j^{n+1/2}-u_j^n, \quad u_j^{n+1/2}=f^{-1}(f_j^{n+1/2}), \end{aligned}$$

which are corrected as follows

$$\begin{aligned} u_j^{n+1}= F\left( {\widetilde{u}}_j^{\,n+1},m_j^{n+1},M_j^{n+1}\right) , \end{aligned}$$

where $$m_j^{n+1}=\min \left( U_{j-1/2}^{n+1},U_{j+1/2}^{n+1}\right)$$ and $$M_j^{n+1}=\max \left( U_{j-1/2}^{n+1},U_{j+1/2}^{n+1}\right)$$.

### The multi-stage embolization optimal regime: modified particle swarm optimization method

To solve the problem of optimal multi-stage embolization, we use a numerical algorithm based on the particle swarm method^25^. To apply this method, the optimal control problem (24)–(26) is rewritten as a functional

40 $$\begin{aligned} {{{\hat{J}}}}_N[q_e]=J_N[q_e]+R \sum \limits _{i=1}^{N} \left( I_1[S_i]+I_2[S_i]\right) , \end{aligned}$$

with penalty functionals $I_1$ and $I_2$, where $I_1[S_i]=0$ when the constraint (25) is met and $I_1[S_i]=1$ when the constraint is violated; similarly, $I_2[S_i]=0$ when constraint (26) is met and $I_2[S_i]=1$ when this constraint is violated; here the solutions $S_i$ depends on the control function $q_e(t)$. In the following calculations, in formula (40) we assume $R=1000$. In the future, the objective functional modification (40) makes it possible to significantly simplify the application of the particle swarm method near a priori unknown part of the boundary domain of control parameters set by the constraints (25) and (26).

The required parameters $\theta _{1i}$, $\theta _{2i}$ and $\gamma _i$, where $i\in \overline{1,N}$, included in the functional (40), are represented as the coordinates of the abstract particle ${\mathbf{x}} \in {\mathbb {R}}^{3N}$, which set ${\mathfrak {M}}=\left\{ {\mathbf{x}}_1,\ldots ,{\mathbf{x}}_M\right\}$ is called as the particles swarm^25,26^. The particle swarm method for minimizing the functional consists in setting the initial position of each particle and organizing the iterative process of moving these particles so that after a given finite number of iterations *I* all the particles end up in a small neighborhood of some point ${\mathbf{x}}_*$ that is the global minimum to the functional (40). At each iteration it is determined the displacement velocity ${{\mathbf{v}}}_j$ and the best position ${\mathbf{p}}_j$ of each particle ${\mathbf{x}}_j$, and also the global best position ${\mathbf{p}}_g$ of all the particles for all the previous iterations. At the end of the iterative process, point ${\mathbf{x}}_*={\mathbf{p}}_g$ is the solution of the minimization problem.

The search for the vector ${\mathbf{x}}_*$ is as follows. First, in the domain of control parameters (31) we allocate *M* bounded disjoint subdomains $\Delta _j$; for each $\Delta _j$ the initial position of single particle is set according to the formula ${\mathbf{x}}^0_j=\Theta [\Delta _j]$, where $\Theta [\Delta _j]$ is a functional that randomly selects the point in the domain $\Delta _j$. The initial velocity of each particle is ${\mathbf{v}}^0_j=0$ and its initial best position is ${\mathbf{p}}^0_j={\mathbf{x}}^0_j$. For all particles ${\mathbf{x}}^0_j$ the value of the functional ${{{\hat{J}}}}_N({\mathbf{x}}^0_j)$ is calculated, after which the formula

$$\begin{aligned} {\mathbf{p}}_g^0=\underset{j=\overline{1,M}}{\mathrm{argmin}}\,{{{\hat{J}}}}_N({\mathbf{x}}^0_j) \end{aligned}$$

is used to find the initial global best position for all particles.

Let the values ${\mathbf{x}}^k_j$, ${\mathbf{v}}^k_j$, ${\mathbf{p}}^k_j$, $j=\overline{1,M}$, and ${\mathbf{p}}_g^k$ be known at the *k*-th step of the iteration. The values at the $(k+1)$-th step of the iteration are determined in several stages.

- The formulas
  $$\begin{aligned} \bar{\mathbf{v}}^{k+1}_j=w^k{\mathbf{v}}^{k}_j+c_1\, r_1^k ({\mathbf{p}}^k_j-{\mathbf{x}}^k_j)+c_2\, r_2^k ({\mathbf{p}}_g-{\mathbf{x}}^k_j),\quad \bar{{\mathbf{x}}}^{k+1}_j={\mathbf{x}}^{k}_j+ \bar{\mathbf{v}}^{k+1}_j \end{aligned}$$
  are used to calculate the preliminary values $\bar{\mathbf{v}}^{k+1}_j$ and $\bar{{\mathbf{x}}}^{k+1}_j$, where $w^k=w_1-w_2k/I$ is the inertia weight^25,26^; $r_1^k$ and $r_2^k$ are the values of random variables uniformly distributed over the interval [0, 1]; $c_1$, $c_2$, $w_1$ and $w_2$ are given positive constants such that $w_2<w_1<1$.
- The coordinates of the particle ${{\tilde{{\mathbf{x}}}}}^{k+1}_j=\left( ({\tilde{x}}_1)^{k+1}_j,\ldots ,({\tilde{x}}_{3N})^{k+1}_j \right)$ are calculated using their preliminary values $\bar{{\mathbf{x}}}^{k+1}_j= \left( ({\bar{x}}_1)^{k+1}_j,\ldots ,({\bar{x}}_{3N})^{k+1}_j \right)$ according to the formula
  $$\begin{aligned} ({\tilde{x}}_l)^{k+1}_j=\left\{ \begin{array}{ll} ({\bar{x}}_l)^{k+1}_j, &{}\quad ({\bar{x}}_l)^{k+1}_j\ge 0, \\ 0, &{}\quad ({\bar{x}}_l)^{k+1}_j<0, \end{array} \right. \quad l=\overline{1,3N}. \end{aligned}$$
  The particle velocities ${{\tilde{\mathbf{v}}}}^{k+1}_j$ are calculated as follows ${\tilde{\mathbf{v}}}^{k+1}_j={{\tilde{{\mathbf{x}}}}}^{k+1}_j-{\mathbf{x}}^k_j$.
- The formulas
  $$\begin{aligned} {\mathbf{p}}^{k+1}_j= & {} \left\{ \begin{array}{ll} {\mathbf{p}}^k_j, &{}\quad {{{\hat{J}}}}_N({\mathbf{p}}^k_j)\le {{{\hat{J}}}}_N({{\tilde{{\mathbf{x}}}}}^{k+1}_j), \\ {{\tilde{{\mathbf{x}}}}}^{k+1}_j, &{}\quad {{{\hat{J}}}}_N({\mathbf{p}}^k_j)>{{{\hat{J}}}}_N({{\tilde{{\mathbf{x}}}}}^{k+1}_j) \end{array} \right. \quad j=\overline{1,M}, \\ {\mathbf{p}}_g^{k+1}= & {}\ \underset{j=\overline{1,M}}{\mathrm{argmin}}\,{{{\hat{J}}}}_N({\mathbf{p}}_j^{k+1}) \end{aligned}$$
  are used to calculate the new best personal and global particles positions.
- The set of all particles ${\mathfrak {M}}$ is randomly divided into two disjoint subsets ${\mathfrak {M}}_1$ and ${\mathfrak {M}}_2$: in the set ${\mathfrak {M}}_1$ the particles are included with a given probability *P*, and in the set ${\mathfrak {M}}_2$ with a probability $1-P$, where *P* is the given number.
- For all $j=\overline{1,M}$, the final positions and velocities of the particles at the $(k+1)$-th iteration are determined by the formulas
  $$\begin{aligned} {\mathbf{x}}^{k+1}_j=\left\{ \begin{array}{ll} \Theta [\Delta _j], &{}\quad {{\tilde{{\mathbf{x}}}}}^{k+1}_j\in {\mathfrak {M}}_1, \\ {{\tilde{{\mathbf{x}}}}}^{k+1}_j, &{}\quad {{\tilde{{\mathbf{x}}}}}^{k+1}_j\in {\mathfrak {M}}_2, \\ \end{array} \right. \quad {\mathbf{v}}^{k+1}_j=\left\{ \begin{array}{ll} 0, &{}\quad {{\tilde{{\mathbf{x}}}}}^{k+1}_j\in {\mathfrak {M}}_1, \\ {{\tilde{\mathbf{v}}}}^{k+1}_j, &{}\quad {{\tilde{{\mathbf{x}}}}}^{k+1}_j\in {\mathfrak {M}}_2, \\ \end{array} \right. \end{aligned}$$

When $k+1=I$ the iterative process is ended.

The proposed algorithm differs from the classical particle swarm optimization method^25^ in that a special initial particle distribution is selected and that at each algorithm iteration the positions of all particles are reinitialized with a certain probability *P*. This modification was required for a more complete examination of the admissible parameter domain and to accelerate the progress of the swarm to an absolute minimum.

### Using clinical data of real patients

To verify the optimal control embolization problem, we use clinical data obtained during monitoring of hemodynamic parameters in process of neurosurgical operations. In these operations, total embolization of the AVM was achieved, that is, the AVM available volume at the operation end was completely filled with the embolic agent. The monitoring was carried out at the National Medical Research Center named after academic Meshalkin^27^ using a Philips ComboMap system and the Philips ComboWire sensor (sensor diameter is 0.36 mm and its length is 1.85 m), which measures blood velocity and pressure inside the cerebral vessels near the pathology. This way the pressure and velocity at the AVM input were obtained before, during and after the embolization, as well as at the AVM output before and after the operation. To obtain the geometrical AVM parameters: the length *L*, the cross-section area *A* as well as input artery cross-section area, data from angiographic studies (perioperative X-ray tomography) were used (Table 1). In numerical calculations, the blood viscosity is $\eta _b=4\, cP$, and the embolic agent viscosity is $\eta _e=18\, cP$, which corresponds to the viscosity of the common embolic agent ONYX18^6^, where *cP* is viscosity unit centipoise.

**Table 1:** Geometrical and filtration characteristics of AVM reconstructed from clinical data.

Patient	*L* (cm)	*A* (cm$^2$)	K (cm$^{2}$)	$k_b(S)$	$p_{*}$ (mmHg)
*K*	2.4	2.275	$7.35 \cdot 10^{-7}$	$S^{1.178}$	80.7
*S*	3	1.327	$8.35 \cdot 10^{-6}$	$S^{1.380}$	114.6
*P*	4.5	4.714	$7.94 \cdot 10^{-6}$	$S^{1.168}$	55

**Figure 2:**
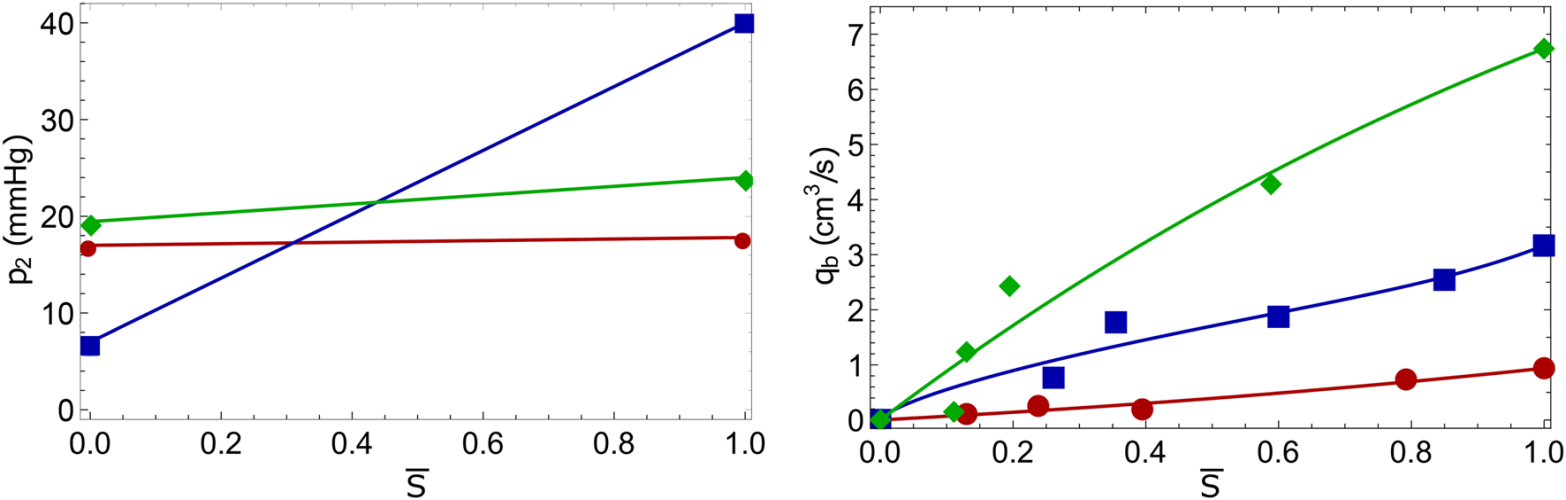
Graphs of functions approximating the clinical data of patients (*K*—circles, *S*—squares, *P*—rhombi): pressure $p_2$ at the AVM output and blood flow $q_b$ at the AVM input, depending on the average blood concentration in the AVM ${\bar{S}}$.

Using the method proposed in the previous papers^22^, the functions $k_b(S)$, $p_2({\bar{S}})$, ${\bar{q}}_b({\bar{S}})$, *f*(*S*) and $r_b(S)$ are constructed for each patient on the basis of clinical data. These functions are included in the initial-boundary value problem for a system of integro-differential equations (18)–(23). In the numerical solution of the optimal control problem (24)–(26), the value $p_*$ is chosen as the maximum pressure at the AVM input during the operation. In calculations without loss of generality, it is assumed that $m=1$, since other constant values of *m* can be reduced to this case by scaling the time variable *t*. The initial cross-section area *A* is assumed to be constant and equal to the average cross-section of the AVM before embolization. It should be noted that the developed method can be used to numerically solve the problem of multi-stage embolization with variable values *m*(*x*) and *A*(*x*). To verify the proposed model, we consider the multi-stage embolization problems for patients *K*, *S* and *P*. The geometrical and filtration characteristics of the pathologies of these patients used for calculations are shown in Table 1 and Fig. 2.

The investigation was conducted in accordance with the Helsinki Declaration and approved by the Inspection Commission of the Meshalkin Clinic (Session No. 549). All included patients gave their informed consent.

## Results

Numerical calculations were performed for patients *K*, *S* and *P* based on the clinical data given above. For the calculations, a space-uniform difference grid was used (32), where $J=100$, with a time step given by the formula (33), in which CFL number $r=0.5$. The small parameter included in the control (28), (29) is $\varepsilon =0.1$ s, which corresponds to the medical practice of quickly stopping the embolic agent supply at the end of each embolization stage. According to the papers^25,26^, the parameters of the particle swarm algorithm were chosen as follows: $P=0.15,\ w_1=0.9,\ w_2=0.5,\ c_1=c_2=1.49$. At each stage, the number of iterations of the particle swarm method is $I=300$.

In order to be consistent with the clinical data, numerical calculations were performed in such a way that the objective functional value was ${{{\hat{J}}}}_N(T_N)\le 0.05$. It was shown that such a functional value for patients *K*, *S* is achieved in two embolization stages, and for patient *P* in three stages. A further increase in the number of embolization stages leads to smaller values of the objective functional. It should be noted that in a real neurosurgical operation, the number of stages can be more than three. To solve the optimal control problem of two-stage embolization by the particle swarm optimization method, the number of particles is chosen to be $M=16$, and for the problem of three-stage embolization, the number of particles is increased to $M=32$. For the optimal regimes obtained as a result of the numerical solution of the multi-stage embolization problem, Table 2 shows the values of the objective functional ${{{\hat{J}}}}_N$ and the corresponding values of the parameters ${\mathbf{p}}_g$ for three patients *K*, *S* and *P*.

**Table 2:** Optimal functional value and optimal control parameters in the case of *N*-stage embolization.

Patient	${{{\hat{J}}}}_N$	*N*	${\mathbf{p}}_g$
*K*	0.0233	2	(18.3616, 4.5428, 0.3262, 2.3975, 2.6563, 1.7723)
*S*	0.0327	2	(2.5994, 0.0000, 0.7303, 0.0000, 0.2459, 2.6093)
*P*	0.0429	3	(7.3273, 6.5195, 0.1412, 1.7879, 6.6116, 0.2083, 0.1527,2.9485, 0.6610)

**Figure 3:**
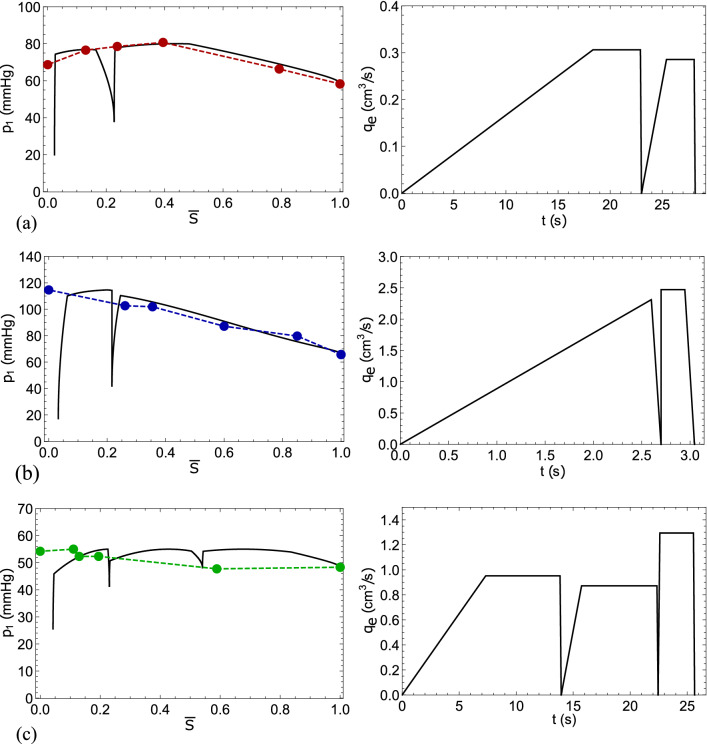
Comparison of the AVM inlet pressures $p_1$ obtained during neurosurgical surgery (circles) and as a result of numerical simulation (solid line), as well as the corresponding optimal embolization regimes $q_e(t)$: (**a**) for patient *K*, (**b**) for patient *S*, (**c**) for patient *P*.

**Figure 4:**
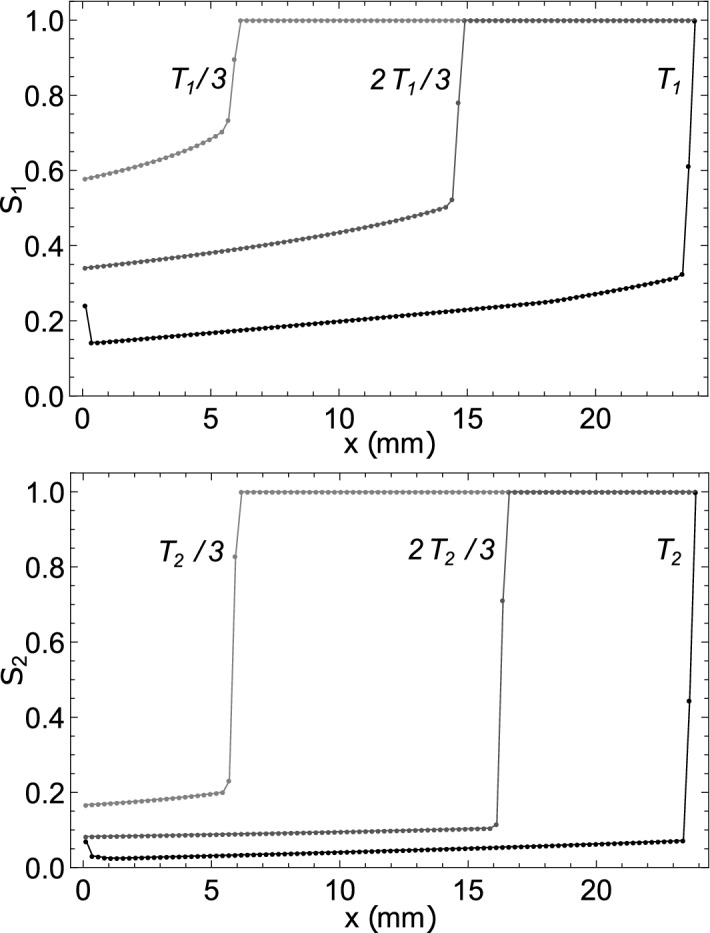
Blood concentrations $S_1(t,x)$ and $S_2(t,x)$ along the AVM for patient *K* in the optimal regime of two-stage embolization.

Figure 3 shows a comparison of the clinical and calculated pressures at the AVM input for the optimal regimes. This figure shows a good correlation between the calculated and clinical pressures for patients *K* and *S*. At the same time, for the patient *P*, the behavior of the clinical and calculated pressures differs to a greater extent. This probably due to the fact that this patient has a difficult clinical case, as indicated by the rupture of AVM vessels after surgery. Thus, it can be assumed that the process of clinical embolization was not carried out in an optimal way for patient *P*. Figure 4 shows the distributions of blood concentration $S_1(t,x)$ and $S_2(t,x)$ along the AVM for the found two-stage optimal embolization regime for patient *K*. For patients *S* and *P*, such distributions have a similar behavior.

## Discussion

Despite the well-developed technique of embolization operations, the risk of perioperative vascular rupture is still a serious danger. In this regard, the development of new methods of mathematical modeling of the AVM embolization process remains an urgent task. For modeling, it is desirable to have detailed information about the AVM geometrical structure, which would allow you to define the functions *A*(*x*) and *m*(*x*). However, modern medical examination methods of reconstructing the AVM geometry, such as computed tomography, magnetic resonance imaging and cerebral angiography, allow us to determine in vivo vessels with an average diameter of at least 0.5 mm. This not enough to determine the detailed geometric structure of the AVM, which often consisting of a large number of conjoined intertwined thin vessels, the diameter of which can reach 0.1 mm. In addition, important information is provided by intraoperative intravascular measurements of hemodynamic parameters near the pathology. Such measurements are possible only in sufficiently large vessels adjacent to the AVM, and measurements directly inside the AVM are seriously difficult and often impossible. Due to the above limitations, the proposed mathematical model is simplified, but despite this, it demonstrates a good agreement between the behavior of the calculated pressure and the clinical one. It should be noted that the question of the theoretical justification of the existence and uniqueness of the optimal control problem solution under consideration requires additional study and can be the subject of another mathematical investigation. Since this theoretical study is not the subject of interest of our applied work, we obtain optimal control using numerical modeling.

On the basis of the mathematical model, it was possible to obtain a good correlation of the calculated and clinical pressure at the AVM input for patients *K* and *S*. Among the considered patients, patient *P* stands out, for which three-stage embolization was required to achieve the functional value ${{{\hat{J}}}}_N < 0.05$, in contrast to patients *K* and *S*, for which such functional values were achieved in two stages. It should be noted that the patient *P* had a hemorrhage immediately after the neurosurgical operation, while the other patients did not have perioperative complications. This suggests that the properties of AVM and its surrounding vessels for patient *P* are significantly different from those of the other two patients. This is also evidenced by a significant difference in the behavior of the clinical and calculated pressure for this patient. It can be hypothesized that the need for a large number of embolization stages indicates a high probability of perioperative complications, and in this case, it is especially useful to find the optimal embolization regime. The appearance of optimal discontinuous controls (“bang-bang” control type) at the last stage for patients *S* and *P* is also interesting.

In the considered model, the influence of surrounding healthy vessels on blood flow through the AVM is taken into account by setting the $q_b$ and $p_2$ functions based on intraoperative measurements near the pathology. Further development of the proposed AVM embolization model may be associated with a more detailed description of the interaction of a healthy vascular network and AVM. This, in particular, will allow us to study the changes in intracerebral blood flow during embolization away from the pathology. It is also important to study the AVM, which consists of several compartments with different properties. To do this, it is necessary to improve the proposed model of optimal AVM embolization in order to take into account more physiological features of the circulatory system. Another important aspect in improving the model may be taking into account the increase in the embolic agent viscosity when it interacts with the blood.

## Conclusion

In this paper it is formulated and investigated the optimal control problem of multi-stage embolization, which arises in the simulation of neurosurgery on pathological blood vessels of the brain. This simulation is performed by describing the joint filtration flow of two phases: blood and embolic agent that thromboses the pathology, as well as taking into account the redistribution of blood into healthy vessels. As a result, an initial-boundary value problem arises for a system of integro-differential equations that includes a hyperbolic divergent partial differential equation with a nonconvex flow function. The numerical solution of this problem, obtained using a monotone modification of the CABARET scheme, is used to solve the optimal control problem of multi-stage embolization by a modified particle swarm method.

In the numerical solution of the considered problem, the parameters and functions included in the system of integro-differential equations and the phase constraints of the optimal control problem are determined on the basis of data obtained for real patients during neurosurgical embolization operations. For the obtained solutions to the optimal control problem of multi-stage embolization, a good agreement between the calculated and clinical pressures is observed. The proposed mathematical model and numerical algorithm will be used to improve the methodology and the safety of neurosurgical operations.
